# Conversational Systems for Social Care in Older Adults: Protocol for a Scoping Review

**DOI:** 10.2196/72310

**Published:** 2025-07-10

**Authors:** Rosiered Brownson-Smith, Ananya Ananthakrishnan, Oksana Hagen, Cen Cong, Amir Aly, Ray B Jones, Edward Meinert

**Affiliations:** 1 Translational and Clinical Research Institute Depth AI Lab Newcastle University Newcastle upon Tyne United Kingdom; 2 Centre for Health Technology Faculty of Science and Engineering University of Plymouth Plymouth United Kingdom; 3 Department of Primary Care and Public Health School of Public Health Imperial College London London United Kingdom

**Keywords:** conversational AI, social care, older adults, usability, social isolation

## Abstract

**Background:**

Social care systems worldwide face increasing demographic and financial pressures. This necessitates exploring innovative technological solutions to enhance service delivery without substantially increasing costs. Conversational interfaces, including interactive voice response, chatbots, and voice assistants, have gained traction as a means to improve accessibility and efficiency in social care. The rapid development of large language models such as ChatGPT has further accelerated interest in conversational artificial intelligence (AI). These technologies can offer intuitive interactions, particularly for individuals with limited digital literacy. However, their real-world impact, usability, and ethical considerations in social care remain underexplored.

**Objective:**

This scoping review aims to synthesize existing literature on the implementation, evaluation, and impact of conversational AI systems within social care settings for older adults. The review will identify best practices, current gaps, and future directions for research and implementation. Key research questions include the following: how are conversational systems implemented on a technical level, and how do older adults and their support systems use them in a social care context? What methods are used to evaluate acceptability, usability, and the impact of broad well-being in the context of older adults’ social care? and What are conversational technologies’ acceptability, usability, and well-being impact in the context of older adults’ social care?

**Methods:**

The review will follow the PRISMA-ScR (Preferred Reporting Items for Systematic Reviews and Meta-Analyses Extension for Scoping Reviews) and Population, Concept, and Context (PCC) frameworks. A systematic search will be conducted across five databases (IEEE, Web of Science, PubMed, ACM, and Scopus) for English-language articles published from 2019 onward. Studies will be included if they empirically examine conversational systems’ implementation, evaluation, or impact for older adults (aged ≥55 years) within a social care context. Two independent reviewers will screen articles and extract data. A descriptive analysis will then categorize findings across key domains such as accessibility, usability, ethical considerations, and well-being outcomes.

**Results:**

The results will be included in the scoping review, which began in March 2025. The analysis is underway and is expected to be completed and submitted for publication by September 2025.

**Conclusions:**

This scoping review will provide an overview of the role of conversational AI in social care, highlighting both opportunities and challenges in implementation. By synthesizing existing research, the review will inform future developments in the use of conversational agents to improve social inclusion, engagement, and well-being among older adults.

**International Registered Report Identifier (IRRID):**

PRR1-10.2196/72310

## Introduction

### Background

Social care services provide essential nonmedical support for older adults [1]. Various deployment approaches (such as community-based care programs, telecare services, and social prescribing) can offer companionship, assistance with daily activities, psychological care, and health-related guidance to older adults. However, social care systems worldwide face growing demographic and financial pressures, spurring the urgent need to leverage technological solutions to expand and improve services without prohibitive cost increases [2,3].

Conversational systems, such as interactive voice response (IVR), chatbots, and voice assistants, have emerged as a promising solution for enhancing efficiencies and broadening access to support within social care [4]. The proliferation of large language models (LLMs) such as ChatGPT has heightened public awareness of conversational artificial intelligence (AI) and accelerated the deployment of chatbots in consumer-facing services and helplines [5]. This trend coincides with evidence that information-intensive fields like health and social care may benefit substantially from voice-based interfaces, which can help streamline and personalize service provision [6,7].

Conversational technologies can simplify interactions with digital systems by offering intuitive communication, particularly for individuals with limited digital literacy or disabilities [8]. Intelligent voice assistants such as Amazon Alexa and Google Assistant have been used to provide medication reminders, offer cognitive stimulation exercises, and facilitate virtual companionship for older adults [9-11]. Similarly, chatbots have been explored to provide mental health support, help alleviate loneliness, and offer emotional assistance to older populations [12,13]. Recent studies indicate that such interfaces foster inclusion and enhance well-being among older adults, empowering them to engage more comfortably with technology [14]. This is especially relevant in settings where reliable online access or smartphone use may be challenging, as telephone-based voice interaction can reduce barriers linked to broadband availability and digital skills [6,15,16]. In social prescribing, which seeks to link individuals with nonclinical community resources that support health and well-being, the integration of voice-based conversational systems has been proposed to improve service accessibility and scale [17].

A broad literature base supports the potential of conversational systems in both health care and business contexts [18-23]. Recent systematic reviews highlight promising outcomes in user satisfaction and preliminary measures of effectiveness [19,20,23]. However, the pace of technological innovation has outstripped robust long-term evaluations of safety, efficacy, and ethical concerns [22]. Beyond technical performance, user experience factors, like trust, privacy, and perceived benefits, remain critical to adoption, particularly in sensitive domains like social care [21]. A recent systematic review of AI conversational agents in health care identified positive or mixed evidence for effectiveness and usability [24]. Still, it highlights that qualitative perceptions of these technologies could be more guarded. Many studies lacked rigorous designs or sufficient reporting, limiting conclusive assessments of their clinical and operational impact.

Given social care’s complex requirements and growing resource constraints, a clear impetus exists to understand how best to use conversational systems at scale. In particular, the natural language capabilities of modern LLMs could further improve voice-based service navigation, automate routine tasks, and connect users to the right resources more efficiently [3,17]. Understanding how conversational systems can be effectively integrated into social care addresses immediate service challenges and informs the broader development of AI-based technologies tailored for sensitive, high-stakes environments.

### Rationale

The existing literature broadly supports the role of digital technologies, including mobile apps, chatbots, and voice assistants, in enhancing well-being among older adults [6,14,25-27]. However, there is a notable gap in synthesized evidence regarding the specific use of conversational agents within social care, a sector already under strain due to an aging population and limited resources [28,29]. While conversational agents are considered accessible and potentially valuable in these settings [19,30], their real-world impact, effectiveness, and acceptability remain insufficiently understood. Questions persist regarding these technologies’ ethical, equitable, and safe deployment in social care. A deeper understanding of their practical implementation, user experience, and broader implications is needed to ensure that they genuinely improve well-being and service accessibility.

While the boundary between social care and health care is often fluid, in this review, social care is viewed as a range of nonmedical services that support individuals’ well-being, independence, and quality of life outside formal health care institutions. Accordingly, this review focuses on community-dwelling older adults rather than those residing in clinical or institutional care settings. Key goals of social care relevant to this population include reducing social isolation and loneliness, which are linked to negative health outcomes and increased mortality [31], as well as promoting autonomy through support for physical activity, nutrition, and chronic disease self-management in nonclinical settings. Additionally, as digital services increasingly mediate access to information and support, addressing digital exclusion among older adults has become an emergent component of social care policy and practice [32]. These domains collectively reflect a broader conception of well-being that extends beyond medical treatment and is increasingly recognized as essential to healthy aging.

This review, therefore, aims to synthesize the emerging literature since 2019 on conversational systems in the age of AI (encompassing IVR, smart speakers, chatbots, and LLM-driven agents) within social care contexts to identify established best practices, current gaps, and future directions for research and implementation. To do this, the scoping review will focus on the following research questions (RQ):

- RQ1: How are conversational systems implemented on a technical level, and how do older adults and their support systems use them in a social care context? 
- RQ2: What methods are used to evaluate acceptability, usability, and the impact of broad well-being in the context of older adults’ social care?
- RQ3: What are conversational technologies’ acceptability, usability, and well-being impact in the context of older adults’ social care?

## Methods

### Ethical Considerations

Ethics approval is not required as data will be obtained from already published sources.

### Overview of the Study Design

The review and search strategy were structured using the PRISMA-ScR (Preferred Reporting Items for Systematic Reviews and Meta-Analyses Extension for Scoping Reviews) [33] and Population, Concept, and Context (PCC) frameworks (Textbox 1) [34].

**Population, Concept, and Context (PCC) framework.**

**Population**

- Older adults (55 years or older)

**Concept**

- Voice assistants, voice interfaces, voice user interfaces, speech-based interfaces, smart speakers, conversational agents, conversational interfaces, chatbots, virtual assistants, interactive voice response

**Context**

- Social care, health and well-being, companionship, engagement

### Search Strategy

Our review will search the following five databases to identify relevant references: IEEE Xplore, Web of Science, PubMed, ACM, and Scopus. This review focuses on technological development and user interaction, so we included computing-focused databases such as the ACM Digital Library and IEEE Xplore. To ensure broader coverage, we searched Web of Science and Scopus as general-purpose literature aggregators and PubMed to capture clinically oriented studies, including formal interventions*.* Relevant terms and keywords were identified for the search based on a preliminary literature examination and previous reviews on related topics. These terms were grouped into 3 themes and were searched by using the following search string structure: voice/conversational agent (abstract) AND older adults (abstracts) (Textbox 2)*.* Given the broad nature of social care, no additional context-specific keywords were included in the search strategy. This approach was intended to maximize sensitivity and ensure that potentially relevant studies were not inadvertently excluded.

**Search strings.**

**Voice/conversational agent**

“voice assistant*” OR “voice interface*” OR “voice user interface*” OR “speech-based interface*” OR “smart speaker*” OR “VUI” OR “conversational agent” OR “conversational interface” OR “conversation agent” OR “chatbot” OR “language interface” OR “virtual assistant” OR “virtual agent” OR “interactive voice response” OR “IVR”

**Older adults**

“older adult*” OR “senior*” OR “elderly” OR “older people” OR “aging” OR “ageing”

### Inclusion Criteria

This study will include empirical research that directly examines the implementation, use, and impact of conversational systems for older adults in a social care context. To ensure relevance to recent advancements in natural language processing, only studies published from 2019 onward will be considered.

To reflect the study’s aims, only papers relevant to the research questions will be included. The research must explore how conversational systems are implemented and evaluated, and their effects on older users, particularly regarding social care and well-being. While studies conducted in health care environments such as hospitals or telemedicine will not be considered, those examining the use of conversational systems for managing long-term conditions, including dementia, will be included, given their relevance to social support and daily living.

The focus will be on research that involves direct engagement between older adults (aged ≥55 years) and conversational systems, providing insights into their practical use and reception. Studies must go beyond theoretical discussions or exploratory interviews, requiring participants to interact with the technology in a real or experimental setting.

### Exclusion Criteria 

Papers that do not describe original research studies, including opinions or editorials, panel discussions, conference summaries, reviews, and meta-analyses, will be excluded, as will papers that present future research plans or study protocols. Retracted articles and papers where the full text is unavailable will also be excluded. Papers that do not evaluate an entire conversational interface with older adults as study participants, such as design or development studies without user evaluation; Wizard of Oz studies without real user interactions; interviews or focus groups conducted without engagement with the technology; and studies assessing only a single feature like voice preferences rather than the overall interaction experience, will not be included.

This review focuses on text- or voice-based conversational systems where interaction occurs through natural language input. Studies involving other interaction modalities, such as smart home systems with Internet of Things integration, robots, digital pets, and avatars, will not be included. Additional interactive modalities can substantially change user experience, which aligns with other studies [35,36]. Systems integrating additional interactive elements (eg, instructional videos) will be considered only if these features do not interfere with the conversational experience.

Examples of excluded systems include smart home systems (eg, Amazon Echo that is used in a wider Internet of Things network of home devices), wearable technologies (eg, smartwatches providing health alerts without interactive dialogue), and embodied systems (eg, robots like Pepper or embodied virtual agents).

Studies evaluating technology explicitly designed for clinical or residential care settings, including hospitals, care homes, and telemedicine applications, will be excluded, as the focus of this review is limited to conversational systems in social care contexts. However, studies investigating home-based management of long-term conditions, such as dementia or Alzheimer disease, will be included if they align with social care objectives rather than medical interventions.

Finally, studies that do not explicitly connect conversational system use to well-being outcomes will be excluded. This includes research that solely examines general patterns of interaction with commercial voice assistants, such as those used for retail or routine daily tasks, unless the study specifically evaluates the system’s impact on social care, inclusion, or well-being. To be included, the system must either be designed with well-being objectives in mind or assessed for its impact on improving well-being in older adults.

### Screening and Article Selection

References will be exported to EndNote 21 (Clarivate), where duplicates will be identified and removed before the screening begins. The screening process will be carried out in two phases: first, titles and abstracts will be reviewed, followed by full-text screening. Two independent reviewers will screen the full texts, with any disagreements resolved through consensus. The reasons for exclusion will be recorded at each stage of screening. The screening process details will be documented in a PRISMA (Preferred Reporting Items for Systematic Reviews and Meta-Analyses) flow diagram to ensure transparency and replicability.

### Data Extraction

Three independent authors will read the full texts of all included papers and extract data into a predetermined table (see Textbox 3 for data extraction items). Any disagreements will be discussed until consensus is reached, and a senior author will be consulted if necessary.

**Details of planned data extraction.**

**Literature**

- Study title
- Author
- Year of publication
- Country of study

**Technology**

- Name of the system
- Does it have a voice interface? (Y/N)
- Purpose of the system as built
- Purpose of the system as evaluated
- Delivered as part of a system? (Y/N)
- How the system was used/delivered (eg, was it prescribed/self-referred)
- Technical implementation information
- Languages offered
- Target population of the system
- Target age group
- Target health condition

**Evaluation**

- Study design
- Main aim
- Study duration
- Sample size
- Sample demographics (age, gender, other)
- Outcomes evaluated
- Validated clinical scales used
- Other validated scales used
- Other (nonvalidated) measures
- Key findings
- How was the system used?

### Data Analysis and Synthesis

A descriptive analysis will be conducted on the extracted data to provide an overview of how conversational systems are implemented and used for older adults in a social care context, how they are evaluated, and how older adults receive them. Depending on the data found in the review, specific analyses related to the outcome measures, technology used, or well-being measures may be conducted. If sufficient data is available, a thematic analysis will be conducted to summarize the qualitative data on the reception of the conversational interfaces by older adults, with subanalyses based on interface type, if applicable.

While we acknowledge the relevance of constructs such as usability, perceived usefulness, and social influence in evaluating technology acceptance, it is important to note that many existing studies on voice AI do not explicitly adopt the Technology Acceptance Model [37] or Unified Theory of Acceptance and Use of Technology [38] frameworks or similar theoretical models. Our primary aim is to outline a broad and inclusive review strategy without restricting the analysis to specific theoretical frameworks at this stage. However, we will remain attentive to these constructs during data extraction and synthesis and, where relevant, will reflect on how the included studies align with or diverge from such models.

In keeping with the methodological guidance for scoping reviews [39], this review will not include formal critical appraisal of included studies. The primary aim is to map the extent, range, and nature of research activity on this topic rather than to assess the effectiveness of interventions or synthesize outcomes in a way that would require a risk of bias assessment. As such, we will not apply formal tools. However, we will extract and report on general methodological characteristics (eg, study design, setting, sample size, data sources), allowing us to comment descriptively on the overall nature and apparent rigor of the evidence base.

## Results

The scoping review began in March 2025, the analysis is underway, and the results will be submitted for publication by September 2025.

## Discussion

### Expected Results

This review aims to synthesize current knowledge about the potential of conversational systems in social care through a systematic analysis of recent medical and technology-related literature. We will examine a wide range of user studies involving older adults across various local and linguistic contexts and with technologies at different stages of development—from early prototypes to fully deployed interventions. These studies will include quantitative and qualitative findings on the impact of such systems on well-being and their usability and acceptability in different applications.

Our goal is to identify relationships and patterns in the data to determine which types of technology (eg, chatbots, IVRs, voice assistants) are most suitable for specific applications, such as addressing loneliness, improving access to health information, or supporting cognitive training. We will also explore how these applications vary across demographic groups, particularly between younger and older segments of the older adult population.

Besides age, older adults differ in education, cognitive and physical ability, mental and physical fitness, technological familiarity, race, cultural norms, and language. To reflect the importance of diversity among older adults, this review will examine how studies report participant demographics, including age distribution, education, physical and cognitive abilities, and language or cultural background. Particular attention will be paid to whether studies acknowledge these differences in their design or analysis.

In addition, we aim to examine the role of modern AI tools, such as LLMs, in the current technological landscape. While these tools offer new opportunities for more natural and engaging user interactions, they also raise significant concerns about data privacy, algorithmic bias, transparency, and trustworthiness. These issues are especially important in work involving vulnerable populations like older adults. These concerns are further compounded by this demographic’s challenges with low digital literacy. Within the scope of this review, we aim to summarize how researchers and developers have approached and addressed these ethical and practical issues.

### Comparison to Prior Work

Recent reviews on conversational technologies for older adults consistently highlight key benefits such as ease of use, improved companionship, and reduced social isolation [40,41]. They also agree on persistent challenges, notably learning barriers, privacy concerns, and technological limitations like speech recognition accuracy [42,43]. Furthermore, a broad consensus exists on the need for more personalized and context-sensitive solutions, especially for users with cognitive impairments [44]. Recent developments in natural language processing, with the emergence and wide proliferation of LLMs, advanced speech recognition, and improved voice synthesis, may solve some of these challenges but also pose additional challenges around privacy and ethics [45].

Additionally, many prior reviews often adopt a narrow focus, concentrating on a specific condition or technology. They demonstrate the value of conversational technologies for social care, IVR systems for data collection and service delivery [46], and smart devices aiding older adults in chronic disease management [47]. Additionally, the use of conversational systems has been examined through several targeted reviews, including telehealth solutions for chronic heart failure [48], behavioral health interventions using voice assistant technologies [23], digital coaching systems for older adults [49,50], and AI-based conversational agents promoting mental health and well-being [51]. Broader reviews provide comprehensive overviews but often concentrate on narrower subsets of literature or singular aspects like design challenges, general trends, user experience, or only commercially available technology [52-55].

We aim to offer an integrative view of recently developed or tested conversational technologies within social care, drawing explicit comparisons across diverse technologies and their practical applications. Our exploration of AI and older systems will contribute to a more in-depth understanding of the unique challenges in implementing them for social care among the older adult population.

### Limitations

This review will only examine original research, meaning relevant opinions, reviews, or other short communications will not be included. As we restrict the finding to the studies with direct interactions between older people and technologies, cross-sectional studies such as population surveys, focus groups, and many participatory method studies that rely on a Wizard of Oz design are not included, despite potential usefulness for understanding such technologies’ context and desirable features. Only texts available in English will be included, neglecting relevant texts in other languages and consequently introducing a potential bias toward English-speaking contexts. Additionally, the review does not include a search of gray literature, meaning it might miss conversational systems developed or deployed that were not featured in academic publishing. Nevertheless, our work will provide a valuable synthesis of existing evidence to guide future research and development.

### Conclusion

This scoping review protocol outlines a systematic approach to mapping the recent literature on the use, evaluation, and reception of conversational systems within social care contexts for older adults. As social care systems worldwide contend with growing demographic pressures, there is an urgent need to understand how digital innovations, particularly voice-based and conversational interfaces, can support well-being, autonomy, and social inclusion among aging populations.

By following the PRISMA-ScR guidelines, this review seeks to identify the technological characteristics and implementation strategies of such systems, as well as how their efficacy, usability, and user experiences are assessed. A key emphasis will be placed on the direct interaction between older adults and these technologies in real or experimental contexts, ensuring that findings reflect actual use and reception rather than hypothetical or design-stage considerations. This review aims to provide critical insights into both the opportunities and challenges associated with these technologies, especially in the fast-paced development of related technologies in the area of natural language processing. In doing so, it will inform future research directions and contribute to developing more accessible, acceptable, and effective conversational systems tailored to the needs of older adults.

## Abbreviations

AI: artificial intelligence
IVR: interactive voice response
LLM: large language model
PCC: Population, Concept, and Context
PRISMA: Preferred Reporting Items for Systematic Reviews and Meta-Analyses
PRISMA-ScR: Preferred Reporting Items for Systematic Reviews and Meta-Analyses Extension for Scoping Reviews
